# Pulmonary function impairment and its relationship with target therapy response in patients with pulmonary arterial hypertension

**DOI:** 10.3389/fmed.2025.1616252

**Published:** 2025-10-14

**Authors:** Xia Xu, Mengshuang Xie, Haijun Li, Junjun Liu, Zhao Yang, Qiushang Ji, Weida Lu, Xiaopei Cui

**Affiliations:** ^1^Department of Geriatric Medicine & Laboratory of Gerontology and Anti-Aging Research, Qilu Hospital of Shandong University, Jinan, Shandong, China; ^2^Xingcheng Community Health Service Center of Xuecheng District, Zaozhuang, Shandong, China; ^3^Department of Cardiology, Qilu Hospital, Cheeloo College of Medicine, Shandong University, Jinan, Shandong, China

**Keywords:** pulmonary arterial hypertension, target therapy, risk status, pulmonary ventilation function, pulmonary diffusion function

## Abstract

**Introduction:**

Patients with pulmonary arterial hypertension (PAH) exhibit exertional dyspnea and decreased exercise capacity, which are not solely attributable to right heart dysfunction. Numerous studies have aimed to elucidate pulmonary function in PH patients and its correlation with disease severity and prognosis; however, the findings remain inconsistent. The impairment of ventilation and diffusion function may partially account for the occurrence of exertional dyspnea in PAH patients.

**Methods:**

This was a single-center prospective observational study. Pulmonary function tests, right heart catheterization, and four-strata risk status stratification were performed in PAH patients. The PAH patients were followed up for 12 months.

**Results:**

A total of 181 PAH patients were enrolled in the study, comprising 62 with idiopathic pulmonary arterial hypertension (IPAH) and heritable PAH (HPAH), 69 with PAH associated with congenital heart disease (CHD-PAH), and 50 with PAH associated with connective tissue disease (CTD-PAH). Forced vital capacity (FVC), forced expiratory volume in 1 s (FEV1), single-breath diffusion capacity for carbon monoxide (DLCO), DLCO% predicted (% pred), and reactance at 5 Hz (X5) were significantly reduced, while residual volume (RV)% pred increased in PAH patients. CHD-PAH exhibited more pronounced ventilation impairment. Six-minute walking distance (6MWD) demonstrated a positive correlation with FEV_1_ (r = 0.353, *p* < 0.01) and FVC (r = 0.373, *p* < 0.01), respectively. A total of 104 patients finished the follow-up. Patients exhibiting FVC% pred values below 82% demonstrated a diminished response to PAH-targeted therapy (OR = 10.553, *p* = 0.000, 95% CI: 2.580–43.165).

**Conclusion:**

PAH patients exhibited impairment in both ventilation and diffusion capacity, while patients with diverse etiologies demonstrated distinct characteristics. FVC and FEV_1_ were positively correlated with 6MWD, respectively. PAH patients with FVC% pred values below 82% demonstrated a diminished response to PAH-targeted therapy.

## Introduction

Pulmonary arterial hypertension (PAH), classified as Group 1 pulmonary hypertension according to ESC/ERS guidelines (1), is a progressive pulmonary vascular disease caused by a variety of etiologies, including idiopathic pulmonary arterial hypertension (IPAH), heritable PAH (HPAH), congenital heart disease-associated pulmonary arterial hypertension (CHD-PAH), and connective tissue disease-associated pulmonary arterial hypertension (CTD-PAH) (1). PAH patients exhibit exertional dyspnea and reduced exercise capacity attributed to right heart dysfunction resulting from increased pulmonary vascular pressure and resistance. However, even after right heart function is improved with optimal PAH-specific treatment, exertional dyspnea remains prevalent among PAH patients, potentially arising from a discrepancy between the neural drive to breathe and the respiratory system’s capacity to respond adequately (2, 3). Mechanical constraints induced by dynamic hyperinflation and excessive ventilatory demand partially elucidate the etiology of exertional dyspnea in patients with PAH who do not exhibit spirometric obstruction (4).

Several studies have aimed to elucidate pulmonary function in patients with pulmonary hypertension (PH) and its association with disease severity and prognosis; however, the conclusions remain inconsistent. Patients with different etiologies exhibit variations in pulmonary function test results. Escribano et al. observed that PAH patients categorized within Venice Groups 1 and 4 demonstrated normal forced vital capacity (FVC), forced expiratory volume in 1 s (FEV_1_), and total lung capacity (TLC) but decreased carbon monoxide transfer factor (TLCO). Furthermore, no correlation was found between pulmonary function and hemodynamics (5). Patients with idiopathic pulmonary arterial hypertension (IPAH) demonstrated not only impaired restrictive lung ventilation, characterized by reductions in FVC and FEV_1_, but also exhibited impaired diffusion function, evidenced by low diffusion capacity of the lung for carbon monoxide (DLCO), which was positively correlated with exercise capacity (6, 7). Patients with CHD-PAH exhibited both restrictive and obstructive ventilatory impairment, and FVC demonstrated a correlation with 6MWD (8, 9). Patients with CTD-PAH demonstrated only mild restrictive abnormality, and those with TLC > 86.11% exhibited a tendency toward improved response to initial combination therapy in the AMBITION study. Furthermore, PAH associated with scleroderma presented higher mortality rates in correlation with decreased DLCO (10, 11).

As recommended by the 2022 European Society of Cardiology (ESC)/European Respiratory Society (ERS) joint guidelines on pulmonary hypertension (PH), the primary treatment goal of PAH is to achieve and maintain a low-risk status, defined as an expected 1-year mortality of less than 5% (12). In the present study, we established a prospective PAH cohort with diverse etiologies, including IPAH, CHD-PAH, and CTD-PAH. Our objectives are to (1) characterize the pulmonary function profiles of PAH patients and (2) further investigate the association between pulmonary function and risk stratification, both at baseline and at follow-up after 12 months of combined PAH-targeted therapy.

## Methods

### Study design and patient enrollment

This single-center prospective observational study was conducted at Qilu Hospital at Shandong University. Healthy volunteers and PAH patients diagnosed by right heart catheterization (RHC), including idiopathic PAH (IPAH), heritable PAH (HPAH), PAH associated with congenital heart disease (CHD-PAH), and PAH associated with connective tissue diseases (CTD-PAH) were recruited from January 2021 to April 2022 with the approval of the Ethics Committee of Shandong University (KYLL-202204-035). Patients with scoliosis, coexisting lung disease, as shown by chest computed tomography (CT) or a history of chronic lung disease, including chronic obstructive lung disease (COPD), interstitial lung disease, asthma, lung cancer, and pneumonia, were excluded. Patients with a smoking index of more than 20 pack-years were also excluded. A total of 100 age-, gender-, and body mass index (BMI)-matched volunteers without pulmonary or cardiac disease were enrolled as control subjects.

### Data collection

Pulmonary function test (PFT), including ventilation, diffusion, and lung mechanics were performed using spirometry (MasterScreen PFT, Jaeger, Germany) for FVC, FEV_1,_ and MEF following ATS/ERS2005 standards (13), Gas Dilution Method for RV and TLC according to ATS/ERS (14), and single breath DLCO for DLCO according to ATS/ERS2017 (15) (MasterScreen Diffusion, Jaeger, Germany). Predicted values were calculated using the Global Lung Function Initiative (GLI) 2012 reference equations (16). Older standards were retained for specific tests due to device compatibility and established clinical protocols at the time of data collection, while ensuring alignment with contemporary reference equations for predicted values. Impulse oscillometry (IOS) (MasterScreen IOS, Jaeger, Germany) for R5, R20, X5, and Fres, following ERS2020 guidelines, was performed (17).

The RHC at rest was performed using a Swan-Ganz catheter for PAH patients. Systolic, diastolic, and mean pulmonary arterial pressure (sPAP, dPAP, and mPAP) were recorded. Cardiac output (CO) was measured by the thermodilution method. Pulmonary vascular resistance (PVR) and cardiac index (CI) were calculated by the standard formula. The World Health Organization functional class (WHO FC), 6-min walking distance (6MWD), right atrial area (RAA), right ventricular diameter (RV), pulmonary artery diameter (PA), tricuspid annular plane systolic excursion (TAPSE), N-terminal pro B-type natriuretic peptide (NT-proBNP), and RHC parameters were collected at baseline. Healthy controls did not undergo 6MWT. The WHO FC was determined by an experienced physician, and the same technician performed transthoracic echocardiography during follow-up visits.

### Risk assessment and follow-up analysis

All patients accepted initial dual therapy, consisting of endothelin receptor antagonists (ERAs, either macitentan or ambrisentan) and phosphodiesterase 5 inhibitors (PDE5is, either sildenafil or tadalafil). NT-proBNP, echocardiography, 6MWT, and WHO-FC were reassessed every 3–6 months. The risk stratification was assessed by a simplified four-strata risk-assessment tool according to the 2022 ESC/ERS PH Guidelines at baseline and at each follow-up for 12 months (12). CHD-PAH patients without defect correction were not included in the follow-up. If low risk status was not achieved within 6 months, further treatment escalation was applied according to guideline suggestion (12). Patients who reached better risk stratification or maintained low risk during follow-up were defined as responders to PAH-targeted therapy; otherwise, they were defined as non-responders.

### Statistical analysis

Analysis was performed with SPSS 23.0 Software (SPSS Inc., Chicago, Illinois). Normal distribution was evaluated using the Kolmogorov–Smirnov test. Continuous variables were presented as the mean with the standard deviation when distributed normally or otherwise as the median with the first and third quartiles (Q1, Q3). Paired t-test, paired rank sum test, or the chi-squared test and Bonferroni correction were used to compare the differences between baseline and follow-up values where appropriate. Ordinal logistic regression analysis, stepwise regression analysis, and ROC curve analysis were performed to evaluate the association between pulmonary function parameters and baseline risk stratification or target therapy response. Significant differences were defined as a *p*-value of < 0.05 (two-tailed test).

## Result

### Characteristics differences between controls and PAH patients

A total of 100 control subjects and 181 PAH patients were recruited. There was no significant difference in age (40.3 ± 9.7 vs. 37.9 ± 12.3 years, *p* = 0.745), gender (female 73% vs. 80%, *p* = 0.171), BMI (23.0 ± 2.4 vs. 23.1 ± 4.3 kg/m^2^, *p* = 0.707), and smoking index (1.1 ± 3.8 vs. 2.9 ± 9.5 pack year, *p* = 0.064) between control subjects and PAH patients (Table 1). PAH patients demonstrated enlarged RAA (23.6 ± 11.8 vs. 16.3 ± 3.4 mm^2^, *p* ***<*** 0.001), RV (34.3 ± 9.3 vs. 23.7 ± 2.8 mm, *p* < 0.001), and PA (30.6 ± 7.2 mm vs. 23.0 ± 2.8, *p* < 0.001) compared to healthy controls.

**Table 1:** Comparison of clinical characteristics, echocardiography and pulmonary function parameters between controls and PAH patients.

	PAH, *n* = 181	Controls, *n* = 100	*t*	*P*
Female, *n* (%)	145 (80)	73 (73)	1.872	0.171
Age, years	37.9 ± 12.3	40.3 ± 9.7	0.326	0.745
BMI, kg/m^2^	23.1 ± 4.3	23.0 ± 2.4	0.376	0.707
Smoking index, pack year	2.9 ± 9.5	1.1 ± 3.8	1.856	0.064
RAA, cm^2^	23.6 ± 11.8	16.3 ± 3.4	6.078	<0.001
RV, mm	34.3 ± 9.3	23.7 ± 2.8	11.085	<0.001
PA diameter, mm	30.6 ± 7.2	23.0 ± 2.8	10.143	<0.001
FVC, L	2.9 ± 0.7	3.9 ± 0.8	10.568	<0.001
FVC% pred	88.4 ± 15.8	102.0 ± 12.1	7.482	<0.001
FEV_1_, L	2.2 ± 0.6	3.3 ± 0.6	13.282	<0.001
FEV_1_% pred	79.1 ± 17.9	99.6 ± 10.9	10.405	<0.001
FEV_1_/FVC	75.8 ± 9.5	84.0 ± 5.2	7.969	<0.001
MEF_75_% pred	80.8 ± 28.4	105.4 ± 17.1	7.887	<0.001
MEF_50_% pred	60.3 ± 26.3	94.3 ± 23.4	10.76	<0.001
MEF_25_% pred	44.3 ± 25.6	83.5 ± 27.0	12	<0.001
RV% pred	123.1 ± 28.9	114.7 ± 32.7	2.154	0.032
DLCO, mmol/min/kPa	6.3 ± 2.5	8.6 ± 1.6	7.939	<0.001
DLCO% pred	73.2 ± 23.6	92.0 ± 13.9	6.836	<0.001
Fres, L/s §	16.1 ± 5.7	14.6 ± 4	1.986	0.049
Z5, kPa/(L/s) §	0.4 ± 0.2	0.4 ± 0.1	1.049	0.296
R5, kPa/(L/s) §	0.4 ± 0.1	0.4 ± 0.1	0.704	0.482
R20, kPa/(L/s) §	0.3 ± 0.1	0.3 ± 0.1	1.025	0.307
X5, kPa/(L/s) §	−0.10 ± 0.1	−0.12 ± 0.1	2.059	0.041
R5-R20% §	24.3 ± 13.6	21.3 ± 12.3	1.54	0.125

BMI, body mass index; RAA, area of right atrium; RV, right ventricular diameter; PA, pulmonary artery; FVC, forced vital capacity; % pred, % predicted; FEV_1_, forced expiratory volume in 1 s; MEF_75_, maximal expiratory flow at 75%, MEF_50_, maximal expiratory flow at 50%, MEF_25_, maximal expiratory flow at 25%; PEF, peak expiratory flow; VC, vital capacity; TLC, total lung capacity; RV, residual volume; DLCO, diffusion capacity for carbon monoxide; Fres, resonant frequency; Z5, impedance at 5 Hz; R5, resistance at 5 Hz; R5-R20, heterogeneity of resistance; X5, reactance at 5 Hz; § partial data missed, *n* = 108.

As shown in Table 1, PAH patients showed decreased FVC (2.9 ± 0.7 vs. 3.9 ± 0.8, *p* < 0.001), FVC% pred (88.4 ± 15.8 vs. 102.0 ± 12.1, *p* < 0.001), FEV_1_ (2.2 ± 0.6 vs. 3.3 ± 0.6, *p* < 0.001), FEV_1_% pred (79.1 ± 17.9 vs. 99.6 ± 10.9, *p* < 0.001), FEV_1_/FVC (75.8 ± 9.5 vs. 84.0 ± 5.2, *p* < 0.001), maximal expiratory flow (MEF)% pred at 75% (MEF_75_% pred, 80.8 ± 28.4 vs. 105.4 ± 17.1, *p* < 0.001), 50% (MEF_50_% pred, 60.3 ± 26.3 vs. 94.3 ± 23.4, *p* < 0.001), and 25% (MEF_25_% pred,44.3 ± 25.6 vs. 83.5 ± 27.0, *p* < 0.001) compared with those of controls. Moreover, 143 (79.0%) PAH patients demonstrated FEV_1_/FVC < 70% whereas all controls demonstrated FEV_1_/FVC ≥ 70%. The single-breath DLCO (6.3 ± 2.5 vs. 8.6 ± 1.6 mmol/min/kPa, *p* < 0.001) and DLCO % pred (73.2 ± 23.6% vs. 92.0 ± 13.9%, *p* < 0.001) of PAH patients were also significantly lower than those of controls. There were 134 (74.0%) PAH patients and 11 (11%) controls with DLCO% pred <80%.

All 108 patients and 100 control subjects completed IOS. There was a significant increase in the resonant frequency (Fres) (16.1 ± 5.7 vs. 14.6 ± 4.0, *p* < 0.05) and peripheral elastic resistance, which was shown by the negative value increase of reactance at 5 Hz (X5) of PAH patients compared with those of controls (−0.12 ± 0.10 vs. −0.10 ± 0.05, *p* = 0.041, as shown in Table 1).

### Pulmonary function comparison among different PAH etiologies

There were 40 patients with IPAH, 22 patients with heritable PAH, 69 patients with CHD-PAH (34 patients with correction operation, including 6 patients with transcatheter intervention operation and 28 patients with surgery) and 50 patients with CTD-PAH (15 patients with systemic lupus erythematosus, 3 patients with systemic scleroderma, 6 patients with Sjogren syndrome, 2 patients with rheumatoid arthritis, 1 patient with aorto-arteritis, and 23 patients with undifferentiated connective tissue disease). IPAH and HPAH were pooled as the IPAH/HPAH group for analysis because they share near-identical clinical phenotypes and treatment responses according to ESC/ERS guidelines (12). Compared to the CTD-PAH or CHD-PAH group, the IPAH/HPAH group showed much higher mPAP (50.0 (39.3, 60.0) vs. 34.5 (29.8, 43.3) vs. 46.0 (32.8, 61.0) mmHg, *p* < 0.001), PVR (9.5 (6.4, 13.0) vs. 5.1 (3.4, 7.7) vs. 5.2 (2.8, 10.1) WU, *p* < 0.001), and lower CI (2.8 (2.3, 3.5) vs. 3.4 (2.8, 4.1) vs. 2.9 (2.4, 4.0) L/min/m^2^, *p* = 0.006) (Table 2).

**Table 2:** Comparison of clinical characteristics, echocardiography, hemodynamics, exercise tolerance and pulmonary function parameters among PAH patients with different etiology.

	IPAH/HPAH, *n* = 62	CHD-PAH, *n* = 69	CTD-PAH, *n* = 50
Female, *n* (%)	54 (87)	48 (70)	43 (86)
Age, years	35.1 ± 10.4	37 ± 11.8	42.6 ± 14.0*
Smoking index, pack year	1 ± 4.6	3.7 ± 10.3	4.2 ± 12.3
RAA, cm^2^	24.7 ± 11.2	25.9 ± 13.7	19.2 ± 7.9#
RV, mm	35.9 ± 9.2	35.3 ± 10.1	30.8 ± 7.3*
PA diameter, mm	28 (25, 31.5)	32 (27, 38)*	28 (25, 31)#
sPAP, mmHg	82 (62.3, 99)	71.5 (48, 91.5)*	53.5 (42.8, 70.3)*#
dPAP, mmHg	33 (26.3, 39)	26.5 (20, 41.3)	21 (18, 27)*#
mPAP, mmHg	50 (39.3, 60)	46 (32.8, 61)	34.5 (29.8, 43.3)*#
PAWP	7.61 ± 2.71	8.0 ± 3.15	8.58 ± 5.35
RAP	5.37 ± 3.34	6.12 ± 3.44	4.14 ± 2.89#
PVR, Wood U	9.5 (6.4, 13.0)	5.2 (2.8, 10.1)*	5.1 (3.4, 7.7)*
CI, L/min/m^2^	2.8 (2.3, 3.5)	2.9 (2.4, 4.0) ※	3.4 (2.8, 4.1)*
6MWD, m	436.8 ± 104.6	457.1 ± 89.9	463.4 ± 78.9
WHO FC (I-II/III-IV)	22/39	43/26*	28/18
NT-proBNP, pg./ml	846.5 ± 848	985.9 ± 2223.6	453.9 ± 821.1
TAPSE, mm	18.1 ± 3.7	17.3 ± 4.5	19.8 ± 3.7
FVC, L	3.1 ± 0.6	2.8 ± 0.8	2.9 ± 0.7
FVC% pred	94.2 ± 15.3	80.5 ± 13.5*	92.1 ± 15.3#
FEV_1_, L	2.5 ± 0.5	2.0 ± 0.6*	2.3 ± 0.6#
FEV_1_% pred	88.1 ± 13.8	67.2 ± 15.7*	84.5 ± 16.3#
FEV_1_/FVC	80.0 (76.1, 84.6)	70.8 (65.0, 77.9)*	78.8 (75.1, 83.2)#
MEF_75_% pred	96.9 (86.5, 106.9)	61.9 (43.4, 79.1)*	91.0 (76.9, 108.3)#
MEF_50_% pred	69.6 (58.7, 85.9)	43.5 (28.1, 53.7)*	72.2 (51.6, 90.5)#
MEF_25_% pred	51.7 (33.8, 68.9)	28.1 (18.8, 37.2)*	46.3 (28.2, 61.1)#
TLC% pred	101.1 ± 12.5	92.1 ± 16.9*	91.4 ± 10.9*
DLCO, mmol/min/kPa	5.6 (4.9, 7.0)	6.8 (5.5, 8.3)*	5.2 (4.1, 5.9)#
DLCO% pred	67.8 (58.9, 77.2)	80.4 (69.4, 97.5)*	63.1 (50.9, 74.6)#
Fres, l/s §	12.8 ± 4.3	19 ± 6.1*	14.7 ± 3.9#
Z5, kPa/(L/s) §	0.4 ± 0.1	0.5 ± 0.2*	0.4 ± 0.1
R5, kPa/(L/s) §	0.3 ± 0.1	0.5 ± 0.2*	0.4 ± 0.1
R20, kPa/(L/s) §	0.3 ± 0.1	0.3 ± 0.1	0.3 ± 0.1
X5, kPa/(L/s) §	−0.1 ± 0	−0.1 ± 0.1	−0.1 ± 0
R5-R20% §	17.2 ± 12.1	30.6 ± 12.9*	21.3 ± 12#
FVC% pred<80%	7 (11.3%)	26 (37.7%)*	8 (16.0%)#
FEV_1_% pred<80%	13 (21.0%)	53 (76.8%)*	16 (32.0%)#
DLCO% pred<80%	43 (69.4%)	29 (42.0%)*	44 (88.0%)#

BMI, body mass index; RAA, area of right atrium; RV, right ventricular diameter; PA, pulmonary artery; sPAP, systolic pulmonary arterial pressure; dPAP, diastolic pulmonary arterial pressure; mPAP, mean pulmonary arterial pressure; PAWP, pulmonary artery wedge pressure; RAP, right atrial pressure; PVR, pulmonary vascular resistance; CO, cardiac output; CI, cardiac index; 6MWD, six-minute walk distance; WHO FC, World Health Organization classification of cardiac function; NT-proBNP, N-terminal pro B-type natriuretic peptide; FVC, forced vital capacity; % pred, % predicted; FEV_1_, forced expiratory volume in 1 s; MEF_75_, maximal expiratory flow at 75%, MEF_50_, maximal expiratory flow at 50%, MEF25, maximal expiratory flow at 25%; PEF, peak expiratory flow; VC, vital capacity; TLC, total lung capacity; RV, residual volume; DLCO, diffusion capacity for carbon monoxide; Fres, resonant frequency; Z5, impedance at 5 Hz; R5, resistance at 5 Hz; R5-R20, heterogeneity of resistance; X5, reactance at 5 Hz. ※partial data missed, *n* = 34. § partial data missed, *n* = 108. After Bonferroni corrected the *P* value, * means difference statistically significant vs. IPAH group using one-way ANOVA, Chi-square test or Kruskal-Wallis test. # means difference statistically significant vs. CHD-APAH group using one-way ANOVA, Chi-square test or Kruskal-Wallis test.

CHD-PAH patients had lowest FEV_1_ (2.0 ± 0.6 L vs. 2.5 ± 0.5 vs. 2.3 ± 0.6, *p* < 0.001), FEV_1_%pred (67.2 ± 15.7 vs. 88.1 ± 13.8 vs. 84.5 ± 16.3, *p* < 0.001), FVC% pred (80.5 ± 13.5 vs. 94.2 ± 15.3 vs. 92.1 ± 15.3, *p* < 0.001), MEF_75_% pred (61.9 (43.4, 79.1) vs. 96.9 (86.5, 106.9) vs. 91.0 (76.9,108.3), *p* < 0.001), MEF_50_% pred (43.5 (28.1, 53.7) vs. 69.6 (58.7, 85.9) vs. 72.2 (51.6, 90.5), *p* < 0.001), and MEF_25_% pred (28.1 (18.8, 37.2) vs. 51.7 (33.8, 68.9) vs. 46.3 (28.2, 61.1), *p* < 0.001) compared to IPAH/HPAH and CTD-PAH patients. There were 26 (37.7%) CHD-PAH patients with FVC% pred <80%, compared to 7 (11.3%) in IPAH/HPAH patients or 8 (16%) in CTD-PAH patients (*p* = 0.001). Similar findings were observed for FEV_1_% pred<80% (76.8% vs. 21.0% vs. 32.0%, *p* < 0.001). In contrast, CHD-PAH patients demonstrated much higher DLCO (6.8 (5.5, 8.3) vs. 5.6 (4.9, 7.0) vs. 5.2 (4.1, 5.9), *p* < 0.001) and DLCO% pred (80.4 (69.4, 97.5) vs. 67.8 (58.9, 77.2) vs. 63.1 (50.9, 74.6), *p* < 0.001) compared to the IPAH/HPAH and CTD-PAH groups (Table 2).

There were significant differences in the Fres, impedance at 5 Hz (Z5), resistance at 5 Hz (R5), and heterogeneity of resistance (R5–R20) among the three groups (Table 2). The peripheral airway resistance, as represented by R5–R20, was significantly higher in CHD-PAH patients than in IPAH/HPAH and CTD-PAH patients (30.6 ± 12.9 vs. 17.2 ± 12.1 vs. 21.3 ± 12.0, *p* < 0.01).

### Correlations between pulmonary function and baseline characteristics

**Figure 1:**
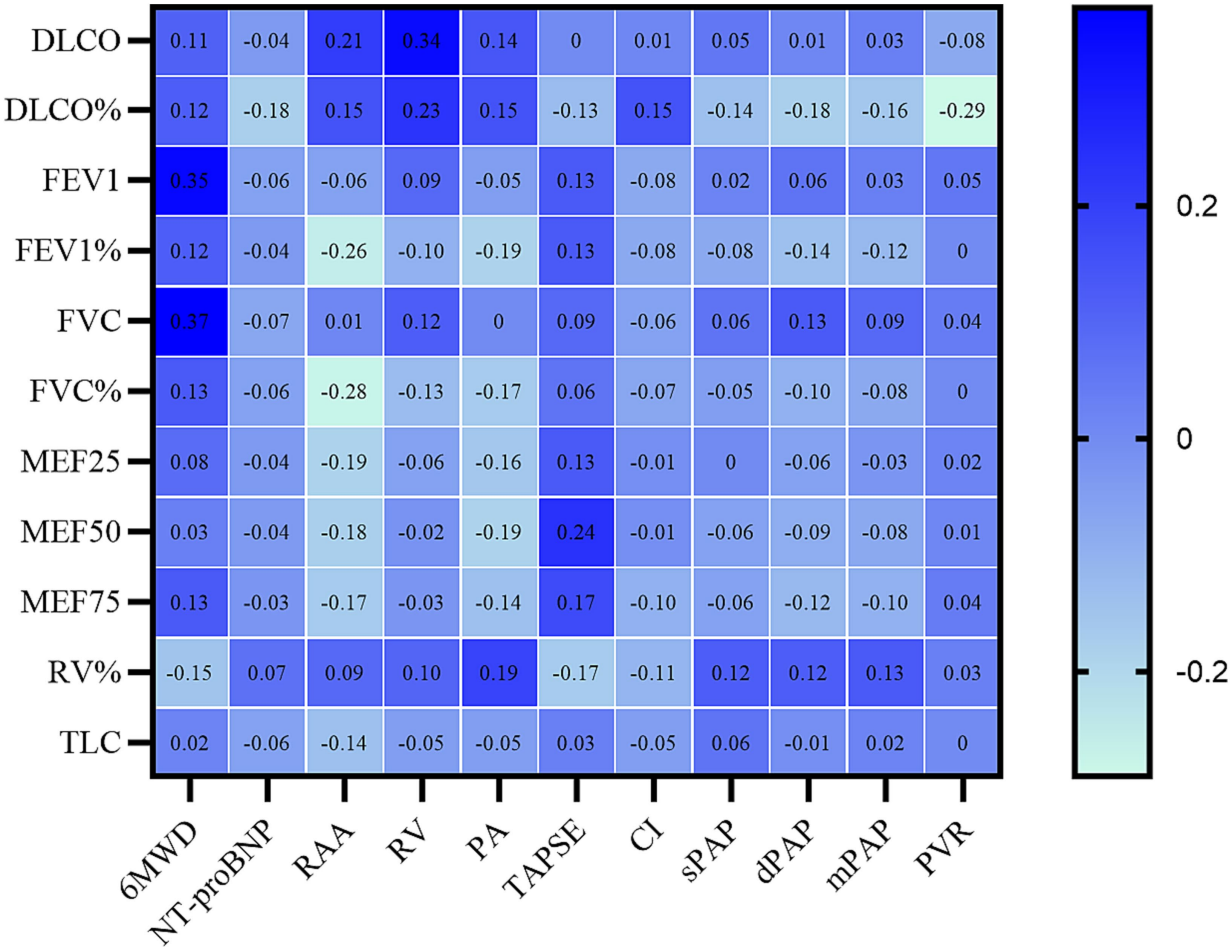
Correlations between pulmonary function test indices and hemodynamic, exercise capacity, and echocardiogram parameters in PAH patients.

As shown in Figure 1, the 6MWD positively related with FEV1 (r = 0.353, *p* < 0.01) and FVC (r = 0.373, *p* < 0.01), respectively. FEV1% pred and FVC% pred were negatively correlated with RA area (Rho = −0.261, *p* < 0.01 for FEV1% pred and Rho = −0.281, *p* < 0.01 for FVC% pred). RV% pred positively correlated with mPAP (Rho = 0.135, *p* < 0.05). DLCO% pred negatively correlated with PVR (Rho = −0.294, *p* < 0.01).

### Relationship between baseline pulmonary function test and target therapy response

A total of 129 patients were enrolled in the follow-up program after excluding 35 uncorrected CHD-PAH patients per protocol, and 17 patients were lost to follow-up before the first visit. Of those 129 enrolled patients, 104 patients completed 1-year follow-up and 25 patients discontinued follow-up prior to study completion. No mortality was observed during this period. None were smokers. A total of 81 patients were classified as responders, while 23 were identified as non-responders. No statistically significant differences in age or sex were observed between these two groups, as shown in Table 3. In comparison to responders, the non-responder group comprised fewer CTD-PAH patients (4.3% vs. 37.1%, *p* = 0.01). However, a significant difference was noted between the etiologies of PAH and baseline risk status. Non-responders also exhibited lower FVC% pred (83.7 ± 12% vs. 94.3 ± 15.8%, *p* = 0.004), FEV1% pred (77.1 ± 13.5% vs. 87.3 ± 16.1%, *p* = 0.008) and TLC% (91.4 ± 8 vs. 98.7 ± 13, *p* = 0.047), as shown in Table 3. Further stepwise logistic analysis considering disease etiology and baseline risk status revealed that FVC% pred (OR = 1.067, *p* = 0.002, 95% CI: 1.024–1.110) was an independent predictor for non-responders. The ROC curve analysis identified 82% as the FVC% pred cutoff point to distinguish non-responders (95% CI: 0.616–0.841). The odds ratio for decreased responsiveness to target therapy in patients with FVC% pred < 82% at baseline was 10.553 (*p* = 0.000, 95% CI: 2.580–43.165), as presented in Table 4.

**Table 3:** Baseline characteristics of target therapy responders.

	Responder, *n* = 81	Non-responder, *n* = 23	*P*
Male, *n* (%)	16 (19.8)	3 (13)	0.462
Age, years	35 (30, 40.5)	30 (23, 42)	0.121
Etiology, *n* (%)			0.01
IPAH/HPAH	39 (48.1)	16 (69.6)	
CHD-PAH	12 (14.8)	6 (26.1)	
CTD-PAH	30 (37.1)	1 (4.3)	
mPAP, mmHg	40 (32, 51.5)	55 (39, 72)	0.004
PVR, Wood U	6 (4.2, 9.6)	12.2 (8.1, 15)	<0.001
CI, L/min/m^2^	3.3 ± 0.9	2.6 ± 0.7	0.002
SvO_2_, %	70.3 ± 7.5	62.4 ± 7.7	<0.001
6MWD, m	471.2 ± 86.4	405.9 ± 108	0.006
WHO FC I/II	53 (65.4)	8 (34.8)	0.008
NT-proBNP, pg./ml	137 (74, 457)	1, 070 (427, 1860)	<0.001
FVC, L	3.1 ± 0.7	2.7 ± 0.6	0.018
FVC% pred	94.3 ± 15.8	83.7 ± 12	0.004
FEV_1_, L	2.5 ± 0.6	2.2 ± 0.6	0.018
FEV_1_% pred	87.3 ± 16.1	77.1 ± 13.5	0.008
FEV_1_/FVC	79 ± 7.9	78.9 ± 6.5	0.939
TLC% pred	98.7 ± 13.4	91.4 ± 8.4	0.047
DLCO% pred	67.7 ± 17.7	67.7 ± 15.2	0.991
DLCO, mmol/min/kPa	5.4 (4.8, 6.4)	5.9 (4.7, 8)	0.301

*P* < 0.05 means the difference statistically significant. mPAP, mean pulmonary arterial pressure; PVR, pulmonary vascular resistance; CI, cardiac index; 6MWD, six-minute walk distance; WHO FC, World Health Organization classification of cardiac function; NT-proBNP, N-terminal pro brfrwedain natriuretic peptide; FVC, forced vital capacity; % pred, % predicted; FEV_1_, forced expiratory volume in 1 s; TLC, total lung capacity; DLCO, diffusion capacity for carbon monoxide.

**Table 4:** Predictors of target therapy non-responder by logistic analysis.

Predictors	Non-responder		
Multivariate	OR	95% CI	*p*
Etiology	2.656	1.054–6.690	0.038
Medium/high risk status at baseline	6.799	1.683–27.460	0.007
FVC% pred < 82%	10.553	2.580–43.165	0.001

OR: odd ratio; CI: confidence interval; FVC, forced vital capacity; % pred, % predicted.

## Discussion

PAH typically exhibits normal or mild restrictive, obstructive, or combined pulmonary function abnormalities (18–20). In the present study, we observed that pulmonary function impairment was associated with different PAH etiologies. CHD-PAH patients demonstrated more severe obstructive ventilation impairment, while CTD-PAH patients exhibited more severe diffusion function impairment, consistent with previous findings (9).

Diffusion capacity of the lung (DL) represents the ability of the lungs to transfer gas from the alveolar space to the red blood cells in pulmonary vessels. Patients with IPAH who are over 50 years of age, male, have a history of smoking, and present with concomitant coronary disease are more likely to exhibit impaired diffusing capacity for carbon monoxide (DLCO) (7). These patients demonstrate reduced exercise performance despite a comparable hemodynamic profile. Farha et al. observed that lung diffusing capacity for nitric oxide (DLNO), but not carbon monoxide (DLCO), decreased in patients with pulmonary arterial hypertension (PAH) over time, indicating a deterioration in the efficiency of the alveolar-capillary unit in PAH (21). Despite exhibiting poorer baseline oxygenation, patients with IPAH and severely reduced DLCO (<43%) demonstrated a comparable response to PAH-targeted therapy as those with moderately reduced or preserved DLCO (22). In the current investigation, we demonstrated impaired DLCO in patients with PAH. Nevertheless, although the DLCO % pred exhibited a negative correlation with PVR, no association was observed with either baseline or follow-up risk status. The differences between our cohort and those previously reported include a relatively younger age, fewer smokers, and fewer coronary comorbidities, which may contribute to the negative DLCO % pred result. Furthermore, the inclusion of PAH patients associated with diverse etiologies may also influence the PFT analysis. The observation that 11% of healthy controls exhibited DLCO% pred values <80%. Several factors may contribute to this finding. First, the inherent technical variability of DLCO measurements, characterized by a higher coefficient of variation (10–15%) compared to spirometry (15), could partially account for these results. Second, undiagnosed comorbidities, such as subclinical emphysema in individuals with smoking exposure (mean pack-year: 1.1 ± 3.8), may have influenced diffusion capacity. Finally, ethnic-specific considerations are relevant; established prediction equations, primarily derived from Caucasian populations, may systematically underestimate DLCO in Asian cohorts, as evidenced by studies in Korean populations (23).

We also evaluated IOS parameters in PAH patients. IOS measures lung resistance at different frequencies to distinguish central and peripheral airway obstruction through regional inhomogeneity. To further differentiate obstruction due to the central airway or the peripheral airway, we conducted IOS analysis. The X5, R5, R5–R20, and R20 represent elastic and interstitial properties, total airway resistance, peripheral airway resistance, and proximal airway resistance, respectively (24). PAH affected lung elasticity, as evidenced by decreased X5. Our integrated analysis of spirometry and oscillometry revealed a distinct pattern of predominantly peripheral, rather than proximal, airway obstruction in CHD-PAH patients. This is evidenced by characteristic reductions in mid-expiratory flows (MEF₇₅, MEF₅₀, MEF₂₅% pred) on spirometry, alongside a significant elevation in R5–R20 (a direct measure of peripheral airway resistance). These convergent data suggest that peripheral, but not proximal, airway obstruction was more prevalent in CHD-PAH patients compared to CTD-PAH and IPAH patients. Although a component of physical lung restriction by enlarged cardiac structures is theoretically possible, the preserved TLC% pred in our CHD-PAH patients argues against it being a primary mechanism. The observed reduction in FVC is more likely a consequence of gas trapping secondary to predominant small airway obstruction.

Abnormal formation or enlargement of vessels can compress the airways and result in small airway obstructions. A close relationship exists between blood vessels and airways throughout lung development (25). Infants with congenital heart disease exhibit an increase in airway smooth muscle and enhanced reactivity (26). The comprehensive remodeling of pulmonary artery hemodynamics in CHD-PAH patients results in more pronounced peripheral airway remodeling.

This study further confirmed that PAH patients had peripheral airway obstruction. A 47-year follow-up of 10,635 patients after congenital heart surgery demonstrated obstructive pulmonary disease as the most common non-cardiovascular morbidity (9%) (27). The possible mechanism includes loss of lung elastic recoil, intrinsic airway narrowing or obliteration, airway inflammation, vasoactive mediators, and mechanical oppression of dilated vessels (8, 13).

In recent times, PAH treatment decisions should be stratified according to disease severity, as assessed through risk stratification. The primary objective is to achieve and maintain a low-risk status, as recommended by clinical guidelines (12). In the present study, we demonstrated that decreased FVC, representing pulmonary ventilation function impairment, resulted in poor risk status improvement after combined target therapy treatment. Two potential explanations are proposed. First, we utilized a four-strata risk-assessment approach, which exhibits increased sensitivity to risk changes from baseline to follow-up (28). This risk assessment tool comprises WHO FC, 6MWD, and BNP/NT-proBNP, with the first two indices reflecting exercise capacity, which is closely associated with ventilation function. Additionally, emerging evidence suggests that lung function is negatively correlated with elevated systemic or paracrine proinflammatory cytokines (29, 30). A close relationship exists between blood vessels and the airways. The pulmonary arteries run alongside and branch from the airways, progressively decreasing in diameter. Perivascular inflammation is a prominent feature in the pathogenesis of PH, and blood cytokine profiles have demonstrated the ability to distinguish PAH immune phenotypes with differing clinical risks independently (31, 32). Consequently, ventilation impairment may deteriorate with the progression of PAH, as demonstrated by Oostveen et al., who observed that FVC decreased by 190 mL/year in PAH patients (17).

## Limitations

There are two major limitations to the study. First, only baseline PFTs, but not PFT changes over time, were included in prognosis analysis. Second, although we did find a relationship between PFT impairment and risk status changes, current results are insufficient for us to conclude whether it is an accompanying phenomenon secondary to PAH progression or a cause of PAH deterioration. A pilot study showed that IPAH patients had significantly increased FEV_1_ and CO with decreased PVR after inhaled salbutamol, a ß2-agonist (33). It is unknown whether patients can benefit from long-term regular bronchodilator therapy. A further randomized study is necessary to go deeply into the use of bronchodilators in PAH patients with ventilation impairment.

## Conclusion

PAH patients had both ventilation and diffusion capacity impairment. The CHD-PAH patients showed apparent peripheral airway obstruction. FVC and FEV_1_ are positively related to 6MWD. PAH patients with FVC% pred <82% showed worse response to PAH-targeted therapy.

## Data Availability

The original contributions presented in the study are included in the article/supplementary material, further inquiries can be directed to the corresponding authors.

## Glossary

BMI: body mass index
CHD-PAH: pulmonary arterial hypertension associated with congenital heart disease
CI: cardiac index
CO: cardiac output
COPD: chronic obstructive lung disease
CTD-PAH: pulmonary arterial hypertension associated with connective tissue disease
DLCO: single-breath diffusion capacity for carbon monoxide
d,s,mPAP: diastolic/systolic/mean pulmonary arterial pressure
ESC: European Society of Cardiology
ERS: European Respiratory Society
FVC: forced vital capacity
FEV_1_: forced expiratory volume in 1 s
Fres: resonant frequency
HPAH: heritable PAH
IOS: impulse oscillometry
IPAH: idiopathic pulmonary arterial hypertension
MEF_75,50,25_: maximal expiratory flow at 75, 50, 25%
NT-proBNP: N-terminal pro B-type natriuretic peptide
PAH: pulmonary arterial hypertension
PEF: peak expiratory flow
PH: pulmonary hypertension
PVR: Pulmonary vascular resistance
RAA: right atrial area
RHC: right heart catheterization
RV: residual volume
R5: resistance at 5 Hz
TAPSE: tricuspid annular plane systolic excursion
TLC: total lung capacity
TLCO: carbon monoxide transfer factor
WHO FC: World Health Organization functional class
X5: reactance at 5 Hz
Z5: impedance at 5 Hz
6MWD: 6-min walking distance
% pred: % predicted.

## References

[1] Simonneau G Montani D Celermajer DS Denton CP Gatzoulis MA Krowka M. Haemodynamic definitions and updated clinical classification of pulmonary hypertension. Eur Respir J. (2019) 53:1801913. doi: 10.1183/13993003.01913-2018, PMID: 30545968 PMC6351336

[2] Mahler DA O'Donnell DE. Recent advances in dyspnea. Chest. (2015) 147:232–41. doi: 10.1378/chest.14-0800, PMID: 25560861

[3] Neder JA Phillips DB O'Donnell DE Dempsey JA. Excess ventilation and exertional dyspnoea in heart failure and pulmonary hypertension. Eur Respir J. (2022) 60:2200144. doi: 10.1183/13993003.00144-2022, PMID: 35618273

[4] Laveneziana P Garcia G Joureau B Nicolas-Jilwan F Brahimi T Laviolette L. Dynamic respiratory mechanics and exertional dyspnoea in pulmonary arterial hypertension. Eur Respir J. (2013) 41:578–87. doi: 10.1183/09031936.00223611, PMID: 22790921

[5] Escribano PM Sanchez MA de Atauri MJ Frade JP García IM. Lung function testing in patients with pulmonary arterial hypertension. Arch Bronconeumol. (2005) 41:380–4. doi: 10.1016/s1579-2129(06)60245-0 16029731

[6] de Almeida GC Pereira MC Moreira MM Souza JRM Paschoal IA. Lung function and stress echocardiography in pulmonary arterial hypertension: a cross-sectional study. Sao Paulo Med J. (2021) 139:505–10. doi: 10.1590/1516-3180.2021.0045.R1.0604221, PMID: 34378739 PMC9632527

[7] Trip P Nossent EJ de Man FS van den Berk IA Boonstra A Groepenhoff H. Severely reduced diffusion capacity in idiopathic pulmonary arterial hypertension: patient characteristics and treatment responses. Eur Respir J. (2013) 42:1575–85. doi: 10.1183/09031936.00184412, PMID: 23949959

[8] Spiesshoefer J Orwat S Henke C Kabitz HJ Katsianos S Borrelli C. Inspiratory muscle dysfunction and restrictive lung function impairment in congenital heart disease: association with immune inflammatory response and exercise intolerance. Int J Cardiol. (2020) 318:45–51. doi: 10.1016/j.ijcard.2020.06.055, PMID: 32634497

[9] Jing ZC Xu XQ Badesch DB Jiang X Wu Y Liu J-M. Pulmonary function testing in patients with pulmonary arterial hypertension. Respir Med. (2009) 103:1136–42. doi: 10.1016/j.rmed.2009.03.009, PMID: 19403296

[10] Kuwana M Blair C Takahashi T Langley J Coghlan JG. Initial combination therapy of ambrisentan and tadalafil in connective tissue disease-associated pulmonary arterial hypertension (CTD-PAH) in the modified intention-to-treat population of the AMBITION study: post hoc analysis. Ann Rheum Dis. (2020) 79:626–34. doi: 10.1136/annrheumdis-2019-216274, PMID: 32161055 PMC7213337

[11] Fisher MR Mathai SC Champion HC Girgis RE Housten-Harris T Hummers L. Clinical differences between idiopathic and scleroderma-related pulmonary hypertension. Arthritis Rheum. (2006) 54:3043–50. doi: 10.1002/art.22069, PMID: 16947776

[12] Humbert M Kovacs G Hoeper MM Badagliacca R Berger RMF Brida M. 2022 ESC/ERS guidelines for the diagnosis and treatment of pulmonary hypertension. Eur Heart J. (2022) 43:3618–731. doi: 10.1093/eurheartj/ehac237, PMID: 36017548

[13] Miller MR Hankinson J Brusasco V Burgos F Casaburi R Coates A. Standardisation of spirometry. Eur Respir J. (2005) 26:319–38. doi: 10.1183/09031936.05.00034805, PMID: 16055882

[14] Wanger J Clausen JL Coates A Pedersen OF Brusasco V. Standardisation of lung volume testing. Eur Respir. (2005) 26:511–22. doi: 10.1183/09031936.05.00035005 16135736

[15] Graham BL Brusasco V Burgos F Cooper BG Jensen R Kendrick A. 2017 ERS/ATS standards for single-breath carbon monoxide uptake in the lung. Eur Respir J. (2017) 49:1600016. doi: 10.1183/13993003.00016-2016, PMID: 28049168

[16] Quanjer PH Stanojevic S Cole TJ Baur X Hall GL Culver BH. Multi-ethnic reference values for spirometry for the 3-95-yr age range: the global lung function 2012 equations. Eur Respir J. (2012) 40:1324–43. doi: 10.1183/09031936.00080312, PMID: 22743675 PMC3786581

[17] Oostveen E King GG Bates J Berger KI Calverley P de Melo PL. Technical standards for respiratory oscillometry. Eur Respir J. (2020) 55:1900753. doi: 10.1183/13993003.00753-2019, PMID: 31772002

[18] Meyer FJ Ewert R Hoeper MM Olschewski H Behr J Winkler J. Peripheral airway obstruction in primary pulmonary hypertension. Thorax. (2002) 57:473–6. doi: 10.1136/thorax.57.6.473, PMID: 12037220 PMC1746348

[19] Sun XG Hansen JE Oudiz RJ Wasserman K. Pulmonary function in primary pulmonary hypertension. J Am Coll Cardiol. (2003) 41:1028–35. doi: 10.1016/s0735-1097(02)02964-9, PMID: 12651053

[20] Alonso-Gonzalez R Borgia F Diller GP Inuzuka R Kempny A Martinez-Naharro A. Abnormal lung function in adults with congenital heart disease: prevalence, relation to cardiac anatomy, and association with survival. Circulation. (2013) 127:882–90. doi: 10.1161/CIRCULATIONAHA.112.126755, PMID: 23382015

[21] Farha S Laskowski D George D Park MM Tang WHW Dweik RA. Loss of alveolar membrane diffusing capacity and pulmonary capillary blood volume in pulmonary arterial hypertension. Respir Res. (2013) 14:6. doi: 10.1186/1465-9921-14-6, PMID: 23339456 PMC3560152

[22] van der Bruggen CE Spruijt OA Nossent EJ Trip P Marcus JT de Man FS. Treatment response in patients with idiopathic pulmonary arterial hypertension and a severely reduced diffusion capacity. Pulm Circ. (2017) 7:137–44. doi: 10.1086/690016, PMID: 28680573 PMC5448550

[23] Hwang YI Park YB Yoon HK Kim T-H Park JH. Development of diffusing capacity prediction equation in healthy Korean adults. J Korean Med Sci. (2010) 25:1120–6. doi: 10.3346/jkms.2010.25.8.1120

[24] Porojan-Suppini N Fira-Mladinescu O Marc M Tudorache E Oancea C. Lung function assessment by impulse oscillometry in adults. Ther Clin Risk Manag. (2020) 16:1139–50. doi: 10.2147/TCRM.S275920, PMID: 33273817 PMC7705955

[25] Hislop AA. Airway and blood vessel interaction during lung development. J Anat. (2002) 201:325–34. doi: 10.1046/j.1469-7580.2002.00097.x, PMID: 12430957 PMC1570917

[26] Schindler MB Bohn DJ Bryan AC Cutz E Rabinovitch M. Increased respiratory system resistance and bronchial smooth muscle hypertrophy in children with acute postoperative pulmonary hypertension. Am J Respir Crit Care Med. (1995) 152:1347–52. doi: 10.1164/ajrccm.152.4.7551393, PMID: 7551393

[27] Raissadati A Haukka J Patila T Nieminen H Jokinen E. Chronic disease burden after congenital heart surgery: a 47-year population-based study with 99% follow-up. J Am Heart Assoc. (2020) 9:e015354. doi: 10.1161/JAHA.119.015354, PMID: 32316818 PMC7428561

[28] Hoeper MM Pausch C Olsson KM Huscher D Pittrow D Grünig E. COMPERA 2.0: a refined four-stratum risk assessment model for pulmonary arterial hypertension. Eur Respir J. (2022) 60:2102311. doi: 10.1183/13993003.02311-2021, PMID: 34737226 PMC9260123

[29] Humbert M Monti G Brenot F Sitbon O Portier A Grangeot-Keros L. Increased interleukin-1 and interleukin-6 serum concentrations in severe primary pulmonary hypertension. Am J Respir Crit Care Med. (1995) 151:1628–31. doi: 10.1164/ajrccm.151.5.7735624, PMID: 7735624

[30] Palma G Sorice GP Genchi VA Giordano F Caccioppoli C D’Oria R. Adipose tissue inflammation and pulmonary dysfunction in obesity. Int J Mol Sci. (2022) 23:7349. doi: 10.3390/ijms23137349, PMID: 35806353 PMC9267094

[31] Voelkel NF Tamosiuniene R Nicolls MR. Challenges and opportunities in treating inflammation associated with pulmonary hypertension. Expert Rev Cardiovasc Ther. (2016) 14:939–51. doi: 10.1080/14779072.2016.1180976, PMID: 27096622 PMC5085832

[32] Sweatt AJ Hedlin HK Balasubramanian V Hsi A Blum LK Robinson WH. Discovery of distinct immune phenotypes using machine learning in pulmonary arterial hypertension. Circ Res. (2019) 124:904–19. doi: 10.1161/CIRCRESAHA.118.313911, PMID: 30661465 PMC6428071

[33] Spiekerkoetter E Fabel H Hoeper MM. Effects of inhaled salbutamol in primary pulmonary hypertension. Eur Respir J. (2002) 20:524–8. doi: 10.1183/09031936.02.02572001, PMID: 12358324

